# RESULTS OF A PILOT IMPLEMENTATION OF AN ADL GUIDELINE FOR NURSING PROFESSIONALS IN THE NETHERLANDS

**DOI:** 10.1093/geroni/igac059.2030

**Published:** 2022-12-20

**Authors:** Svenja Cremer, Michel Bleijlevens, Silke Metzelthin, Janneke de Man-van de van Ginkel, Sandra Zwakhalen

**Affiliations:** Maastricht University, Maastricht, Limburg, Netherlands; Maastricht University, Maastricht, Limburg, Netherlands; Maastricht University, Maastricht, Limburg, Netherlands; Julius Center for Health Sciences and Primary Care, Utrecht, Utrecht, Netherlands; Maastricht University, Maastricht, Limburg, Netherlands

## Abstract

Nursing care in activities of daily living (ADL), such as washing, dressing, or eating is frequently provided while being poorly informed by scientific evidence. To address nursing professionals’ need for guidance we developed a practice guideline on ADL-care. This guideline comprises eleven core recommendations on involving care receivers and informal caregivers in ADL-care, identifying ADL-care needs, and effective ADL-interventions. Since the success of this guideline hinges on its actual use by nursing professionals, we assessed the use and determined influencing factors to guide targeted strategies for future implementation in different nursing care settings. In a mixed-method study, nursing professionals documented the number of core recommendations applied over three weeks using recording forms and a self-administered questionnaire to identify barriers and facilitators. In addition, we conducted focus groups to capture team experiences in applying the 11 core recommendations and clarifying survey results. Seven nursing care teams participated from various settings: hospital (n=1), rehabilitation (n=2), home-care (n=1), and long-term care (n=3). Preliminary results reveal that participants consider the core recommendations compatible with and adaptable to current practices, and their care settings. Participants experienced advantages over existing practices. However, core-recommendation usage and perception of influencing factors appeared to be highly context-dependent, especially regarding the involvement of informal caregivers in ADL-care. Limited knowledge on the application of interventions to improve ADL limited the use of some recommendations. Our results underline the necessity of careful selection of targeted strategies for each setting tailoring the core recommendations to the different qualifications and roles of nursing professionals.

